# A Novel Non-invasive Index of Cardiopulmonary Reserve for the Prediction of Failure of Weaning From Mechanical Ventilation

**DOI:** 10.7759/cureus.27150

**Published:** 2022-07-22

**Authors:** George T Nikitas, Stylianos Kykalos, Evangelia Ntikoudi, Ioannis Vasileiadis, Antonia Koutsoukou, Nikolaos I Nikiteas

**Affiliations:** 1 Cardiology, Panarkadian General Hospital of Tripolis, Tripolis, GRC; 2 General Surgery, 2nd Department of Propaedeutic Surgery, Laiko Hospital, Athens Medical School, National and Kapodistrian University of Athens, Athens, GRC; 3 Intensive Care Unit, Sotiria Thoracic Diseases Hospital, Athens, GRC; 4 Respiratory Medicine, 1st Department of Respiratory Medicine, National and Kapodistrian University of Athens, Athens, GRC; 5 Internal Medicine, National and Kapodistrian University of Athens, Athens, GRC; 6 Hellenic Minimally Invasive and Robotic Surgery (MIRS) Study Group, 2nd Department of Propaedeutic Surgery, Laiko Hospital, Athens Medical School, National and Kapodistrian University of Athens, Athens, GRC

**Keywords:** extubation failure, medical intensive care unit (micu), weaning trial, weaning failure, prediction index, respiratory exchange ratio

## Abstract

Purpose: To develop an easy-to-implement prediction index of weaning failure for ICU patients.

Materials and methods*: *We developed a prediction index modifying respiratory exchange ratio (RER), Mod-RER, a parameter measured during the cardiopulmonary exercise test (CPET) based on respiratory quotient. The Mod-RER index is the ratio of partial pressure of CO_2_ in central venous blood over the difference of partial pressure of O_2_ in arterial and central venous blood (Mod-RER=PcvCO_2_/PaO_2_-PcvO_2, _where PcvCO_2_ = partial pressure of CO_2_ in central venous blood, PaO_2 _= partial pressure of O_2_ in arterial blood, and PcvO_2_ = partial pressure of O_2_ in central venous blood). We prospectively tested its predictive value, compared to other indices of weaning outcome, in an observational study of difficult-to-wean ICU patients.

Results:Mod-RER index increased significantly only in failed trials and receiver operating characteristic (ROC) analysis for prediction of outcome based on Mod-RER index change had an area under the curve (AUC) 0.80 (*p*<0.001). Mod-RER change exhibited the highest sensitivity (84.6%) and specificity (78.1%) among the tested indices, with the optimal cut-off of 19.3%. Comparison of AUCs did not reach statistical significance (*p*=0.106).

Conclusions: We conclude that Mod-RER index is an accurate, easy-to-use prediction tool of weaning failure, useful in decision making of timely extubation of ICU patients, especially in the demanding era of the coronavirus disease 2019 (COVID-19) pandemic.

## Introduction

Difficulty to wean from mechanical ventilation is a common problem for 26-42% of intensive care unit (ICU) patients. Pathophysiology of difficult weaning is complex, including cardio-respiratory failure and muscle fatigue, especially of the diaphragm- usually on the basis of ICU-acquired weakness [1,2].

Weaning failure is termed as failure to pass a spontaneous breathing trial by clinical terms or the need for re-intubation within 48 hours [1]. Although delay of extubation is a known predisposing factor for pneumonia and an increase in ICU mortality [3], re-intubation after a failed extubation carries a high risk for aspiration and high mortality as well [4].

Several weaning protocols have been tested during the past decades. Previous studies showed that spontaneous breathing trials through a T-piece, either once or more times daily, resulted in faster weaning and established this technique as the most effective and popular among intensive care physicians [5]. 

During the past 35 years, several prediction models and indices have been developed to predict the outcome of an extubation. Yet, so far, none of them has been proven both accurate enough and easy to implement in everyday practice so as to be considered a “gold standard” in the decision making process of weaning and extubation.

## Materials and methods

Developing a novel prediction index

Cardiopulmonary exercise testing (CPET) provides a wide range of information on the performance capacity of the heart, lungs, vasculature, and blood to sustain oxygen delivery and the removal of carbon dioxide from the muscles by measurements of the inhaled oxygen (O_2_) and the exhaled carbon dioxide (CO_2_) and parameters of respiratory function [6]. The evaluation of an ICU patient with CPET prior to weaning seems impossible. Yet, if we consider a weaning trial as an exercise “analogue” for ICU patients, indices of CPET could be adapted for the evaluation of the patient’s effort, muscle fatigue, cardiorespiratory reserve, and Respiratory exchange ratio (RER) seems an ideal “candidate”. RER is the ratio of exhaled CO_2_ (VCO_2_) above the oxygen uptake (VO_2_) (RER = VCO_2_ /VO_2_). At rest, in normal subjects, values range between 0.7 and 1.0 depending on the subject’s diet and substrate use during aerobic metabolism, being by virtue the equivalent of respiratory quotient [7]. During exercise though, the increased metabolic demands lead to hyperventilation, increased CO_2_ production, and, above the anaerobic threshold, lactic acid buffering that contributes to the multiplication of CO_2_ output and increases the numerator faster than the denominator. Values of peak RER above 1.0 correlate well with the excess of the anaerobic threshold during exercise [8].

Calculation of RER during exercise is quite simple, providing that one can measure volumes of inhaled O_2_ and exhaled CO_2_. In a weaning trial of a patient connected to a respirator such measurements could be easy, yet for a T-piece trial, which is the mainstay in weaning, this would be practically impossible. On the contrary, in the ICU setting, it is much easier to analyze blood samples. Measurements of veno-arterial CO_2_ concentration difference, arterial-venous O_2_ concentration difference, and calculation of their ratio, could provide us with an appropriate analog for a modified version of RER. Yet, such calculations are again complex and rely on a variety of parameters including hemoglobin, pH, and temperature, and, particularly for venous CO_2_ concentration, there are questions about the accuracy of its calculation during exercise [9].

We attempted an adaptation of RER index in a simplified form utilizing partial pressures values of O_2_ and CO_2_ from arterial and venous blood samples, making two assumptions based on previous physiology studies: (i) First, the exhaled CO_2_ in the numerator would be proportional to the partial pressure of CO_2_ in the central venous blood sample, and (ii) second, that the oxygen uptake in the denominator would be proportional to the arterial-venous difference of partial pressure of O_2_.

We already know that partial pressure of mixed venous CO_2_ (PvCO_2_) is proportional to the VCO_2_ [10]. The apparent question on the implementation of PvCO_2_ instead of the veno-arterial CO_2_ difference (PvCO_2_-PaCO_2_) as an indicator of CO_2_ production and exhalation (taking the place of VCO_2_ in the ratio) is answered by previous data suggesting that the partial pressure of arterial CO_2_ (PaCO_2_) remains stable during exercise [11] in contrast with PvCO_2_ and VCO_2_that steadily increase during exercise, to a total degree of two times and 16 times, respectively, from rest to peak exercise [9].

VO_2_ in the denominator could be calculated using the Fick equation but calculations are particularly complex for an “easy-to-use” index. It increases 10 to 20-fold during exercise in healthy individuals, but in patients with heart failure (a usual pathology in ICU patients, either chronic or acute due to ischemia, septic cardiomyopathy, etc.) this increase is reduced by two-thirds and relies primarily on arterial-venous oxygen content difference [12]. 

During exercise, the muscle oxygen uptake increases and this increase is inversely proportional to capillary O_2_ pressure (which equals the arterial-venous partial pressure of oxygen (PO_2_) difference). So, the more oxygen a muscle consumes, the bigger the arterial-venous PO_2_ difference grows, driven by the reduction of capillary PO_2_. This phenomenon continues until the capillaries reach their oxygen diffusion limit (at the levels of the “critical capillary O_2_ pressure” of 15-20mmHg), leading to the advent of anaerobic metabolism in order to cope with the continuously increasing muscle energy demands. So we suppose that the arterial-venous PO_2_ difference (PaO_2_-PvO_2_) will be an analog of muscle oxygen uptake, at least before the advent of anaerobic metabolism.

Finally, we know that the measurements of partial pressures of O_2_ and CO_2_ in central venous blood can replace those in mixed venous blood, as proven in situations like the evaluation of tissue hypoxia in septic shock models [13].

So the suggested modified index (Mod-RER) that we propose could be:

Mod-RER = PcvCO_2_/PaO2-PcvO_2_, where PcvCO_2_ = partial pressure of CO_2_ in central venous blood, PaO_2 _= partial pressure of O_2_ in arterial blood, and PcvO_2_ = partial pressure of O_2_ in central venous blood.

Studies regarding blood partial pressures of O_2_ and CO_2_ during exercise showed that indeed PvCO_2_ is steadily increased, both before and after the anaerobic threshold, following the increase in CO_2_ production and expulsion [10]. PaO_2_ is slightly increased immediately at the beginning of exercise [11] and as the effort escalates towards the maximum, the PaO_2_ values return close to the rest levels [14]. And finally, PvO_2_ values do decrease during exercise [15,16] stabilizing at the lowest levels before the advent of anaerobic metabolism without further reduction [17].

So, if we monitor the Mod-RER index during a weaning trial in ICU patients, we expect that in patients that overcome the work of breathing without significant distress, Mod-RER index won’t change significantly, since both the numerator and the denominator will increase concomitantly. In those that cannot cope with the work of spontaneous breathing, the incoming anaerobic metabolism and a further increase in CO_2 _production will lead to a significant rise of PcvCO_2_, while the denominator of the ratio (PaO_2_-PcvO_2_) will stabilize and so Mod-RER values will probably increase during the trial, indicating the lack of additional functional reserve for successful extubation.

Study design and data acquisition

This was a prospective single-center observational cohort study conducted in a seven-bed medical-surgical ICU of a tertiary hospital, Hippokrateion General Hospital of Athens, Athens, Greece. The study was approved by the Ethics Committee of Hippocrateion General Hospital of Athens, Athens, Greece (approval number ES68572015). Because of the observational nature of the study, patients’ consent was waived. Eligibility criteria included duration of intubation and mechanical ventilation of more than 24 hours, a pair of arterial and central venous blood samples drawn for a full blood gas analysis, including Lac concentration, before and at the end of the weaning trial, and age above 18 years. Exclusion criteria were non-reversible coma or vegetative state of the patient.

We prospectively monitored patients planned for a T-piece trial during weaning from mechanical ventilation without any intervention in the process. The treating physician’s decision to extubate lies only on clinical judgment and bedside measurements of arterial and venous blood samples (arterial pH, partial pressures of O_2_ and CO_2_, central venous oxygen saturation (ScvO_2_), etc.). Failure of the weaning trial was determined by clinical criteria (tachypnoea, tachycardia, hypertension, and desaturation) verified by bedside tests if necessary. Extubation was considered successful if the patient did not require mechanical ventilation (invasive or not) within the next 48 hours [1].

In order to calculate the Mod-RER index, and since the trial was non-invasive, we had to restrict our study population only to T-piece trials in which the treating physician had decided to examine arterial and venous blood samples for gas measurements before and at the end of the trial. This is current practice in our ICU for the evaluation of PaO_2_/fraction of inspired oxygen (FiO_2_), pH, and ScvO_2_ [1,18]. The duration of the trials varied from minutes to several hours, with most of them ranging between 30 minutes to two hours. The oxygen flow rate during the test was not stable but was adjusted depending on the patients’ oxygen saturation as measured by blood analysis (SaO_2_) with a target between 92% and 96% and the interruption criteria were clinical (including tachypnoea, tachycardia, and persistent desaturation). The first samples are drawn before the beginning of the trial (always on pressure support ventilation) and the last two at the end, before the decision whether to extubate the patient or to reinstitute mechanical ventilation. Arterial samples are drawn from previously placed arterial catheters in the radial or femoral artery and venous samples from central catheters introduced via subclavian or jugular vein sheaths and positioned within the superior vena cava (SVC) at the level of the carina or just above the cavo-atrial junction (the junction of the SVC and right atrium). A radiographic verification took always place. All blood samples during the study were collected in plastic syringes containing a small amount (0.05-0.10 ml) of heparin solution (5.000 IU/ml) and analyzed using a Cobas b121 blood gas analyzer (F. Hoffmann-La Roche AG, Basel, Switzerland) either immediately or within one hour, if kept in a standard freezer (-18oC).

Using exclusively the abovementioned sample measurements, we also tested other indices that might help predict the outcome of weaning and required no other examinations (e.g. hemodynamic measurements, systemic and pulmonary pressures, lung function data, respiratory frequency, and minute ventilation). We measured changes in arterial pH, PaCO_2_, ScvO_2_, lactic acid concentrations, arterial-venous oxygen difference (a-vO_2_ difference), oxygen extraction ratio in its simplified form, (SaO_2_-ScvO_2_)/SaO_2_, and veno-arterial carbon dioxide tension difference over the arterio-venous oxygen content difference, [P(v−a)CO_2_]/[C(a−v)O_2_], the last three with central venous instead of mixed venous values). The choice to study these particular indices was based either on previous studies exploring parameters of successful weaning [18,19,20] or the information they provide regarding respiratory and metabolic tissue demands and the induction of anaerobic metabolism [19,21].

Statistical analysis

Variables with approximately symmetric distributions were summarized as mean and standard deviation (SD), variables with skewed distribution as the median and interquartile range (IQR), and qualitative variables as absolute and relative frequencies. For comparisons of proportions, chi-square and Fisher’s exact tests were used. Student’s t-tests were computed for comparison of mean values when the distribution was approximately symmetric and the Mann-Whitney test, when the distribution was not approximately symmetric. Differences in changes of study variables during the follow-up between the two study groups (as defined from failure and success) were evaluated using repeated measurements analysis of variance (ANOVA). Receiver operating characteristic (ROC) analysis and area under the curve (AUC) was used to evaluate the predictive ability of study variables that their change was significantly associated with the outcome. Sensitivity (SE) and specificity (SP) values were determined for optimal cut-offs and were evaluated using the Youden index [22]. In order to investigate evidence of difference in predictive ability of different indices, AUCs were calculated and compared using the Wald test of the null hypothesis that all AUC values are equal [23]. Logistic regression analysis in a stepwise method (p for entry 0.05, p for removal 0.10) was used in order to find independently associated factors with the outcome. Adjusted odds ratios (aOR) with 95%CI were computed from the results of the logistic regression analyses. All p-values reported are two-tailed. Statistical significance was set at 0.05 and analyses were conducted using Stata Statistical Software: Release 11 (2009; StataCorp LP, College Station, Texas, United States).

## Results

**Figure 1:**
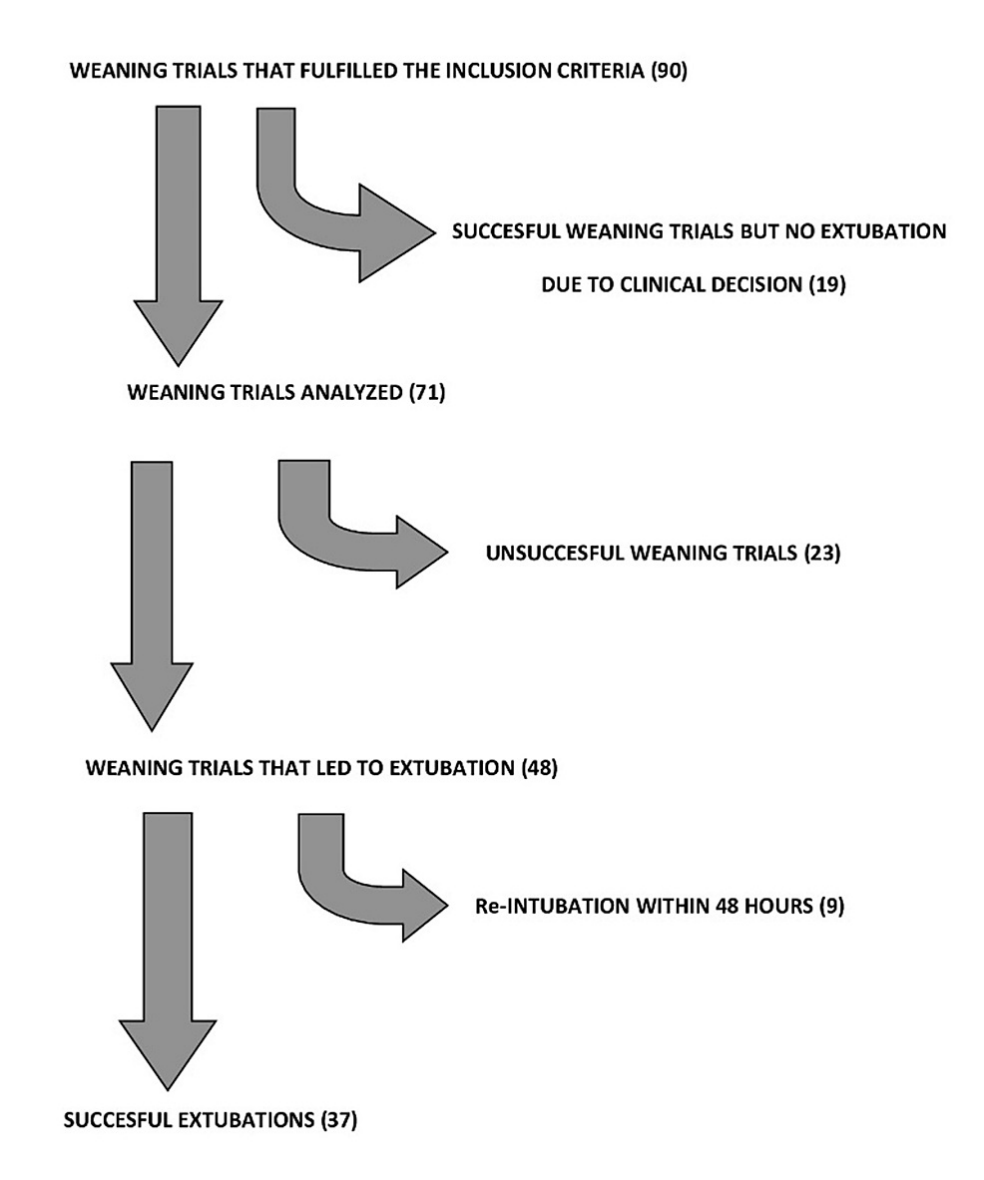
Distribution of the monitored weaning trials during the study period

We monitored all the weaning trials conducted in our ICU from December 1, 2013, to May 31, 2015. Data were collected from a total of 90 T-piece trials in 53 patients that fulfilled the prespecified eligibility criteria. The trials that, although considered successful by the treating physician, did not lead to extubation due to other reasons (poor cooperation of the patient, impairment of mental status during the trial, etc.) were excluded. Finally, the analysis included 71 trials in 51 patients (33 men, 64.7%, mean age 68.4 years (SD=14.4 years)) with 48 extubations and 23 unsuccessful trials where mechanical ventilation was reinstituted. Nine of the extubated patients had to be re-intubated within 48 hours, raising the total number of failed trials to 32 (45.1%). The distribution of the monitored trials is depicted in Figure 1.

 The characteristics of the trials according to outcome are depicted in Table 1.

**Table 1:** Sample characteristics in total trials and according to outcome +Pearson’s chi-square test; ++Fisher’s exact test; ‡Student’s t-test; ‡‡Mann-Whitney test CVVHD: continuous veno-venous hemodialysis, APACHE II: acute physiology and chronic health evaluation II

		Outcome		
	Total sample (N=71; 100%)	Failure (N=32; 45.1%)	Success (N=39; 54.9%)	
	N (%)	N (%)	N (%)	P
Sex				
Males	41 (57.7)	17 (53.1)	24 (61.5)	0.475+
Females	30 (42.3)	15 (46.9)	15 (38.5)	
Age, mean (SD)	70.8 (13.9)	74.5 (11.1)	67.7 (15.3)	0.038‡
Days of intubation, median (IQR)	8 (4-22)	21.5 (7.5-37)	5 (1-12)	<0.001‡‡
Trial duration (min), median (IQR)	80 (50-240)	102.5 (60-275)	75 (45-140)	0.340‡‡
APACHE II, mean (SD)	22.3 (8.3)	23.4 (8.4)	21.4 (8.2)	0.312‡
Chronic respiratory failure	25 (35.2)	15 (46.9)	10 (25.6)	0.062+
Cardiac failure	30 (42.3)	15 (46.9)	15 (38.5)	0.475+
Stroke	11 (15.5)	8 (25)	3 (7.7)	0.055++
Dementia or anoxic-ischemic encephalopathy	7 (9.9)	6 (18.8)	1 (2.6)	0.041++
ICU-acquired weakness	24 (33.8)	15 (46.9)	9 (23.1)	0.035+
CVVHD	2 (2.8)	1 (3.1)	1 (2.6)	1.000++
Tracheostomy	17 (23.9)	16 (50)	1 (2.6)	<0.001+
Death	16 (22.5)	13 (40.6)	3 (7.7)	0.001+

The failed weaning trials were in significantly older patients with a longer duration of mechanical ventilation and more frequently in patients with dementia or anoxic-ischemic encephalopathy, ICU-acquired weakness, or tracheostomy. Eleven patients died in the total sample of 51 (21.6%) and the proportion was significantly greater in those that did not have successful extubation (45% vs. 6.5%, p=0.001).

Changes in study parameters during the trials according to outcome are shown in Table 2. PaCO_2_ had a significant mean increase of 11.08% in failed trials and a significant mean increase of 4.48% in successful ones. The overall change was significantly different between the two study groups as indicated by the significant interaction effect of the analysis. ScvO_2_ decreased significantly only in failed trials, while Mod-RER increased significantly only in failed ones. For both ScvO_2_ and Mod-RER, the significant interaction effect indicated a significantly different change between the two study groups. Also, at post measurements, ScvO_2_ levels were lower in those that failed while the Mod-RER measures were lower in those that succeed.

**Table 2:** Changes in study parameters during follow-up according to outcome _1_ p-value for group effect; _2 _p-value for time effect; _3_ Effects reported include differences between the groups in the degree of change (repeated measurements ANOVA) PaCO2: partial pressure of carbon dioxide in arterial blood; ScvO2: central venous oxygen saturation, Mod-RER: modifying respiratory exchange ratio index

		Pre	Post	Change %		
	Outcome	Mean (SD)	Mean (SD)	Mean (SD)	P_2_	P_3_
pH	Fail	7.42 (0.04)	7.39 (0.05)	-0.48 (0.63)	0.752	0.455
	Success	7.44 (0.05)	7.29 (0.85)	-1.99 (11.41)	0.146	
	P_1_	0.103	0.520			
PaCO_2_	Fail	39.33 (7.06)	43.97 (10.49)	11.08 (11.28)	<0.001	0.006
	Success	38.88 (6.33)	40.48 (7.12)	4.48 (10.59)	0.031	
	P_1_	0.778	0.101			
ScvO2	Fail	67.08 (7.84)	65.05 (8.26)	-2.81 (8.59)	0.049	0.016
	Success	68.28 (7.23)	69.90 (7.33)	2.91 (10.79)	0.109	
	P_1_	0.503	0.011			
Lac	Fail	1.31 (0.46)	1.29 (0.45)	1.28 (22.8)	0.684	0.555
	Success	1.49 (0.84)	1.51 (0.82)	3.07 (16.66)	0.667	
	P_1_	0.270	0.176			
P(v − a)CO^2^/C(a − v)O_2_	Fail	1.79 (0.97)	1.65 (0.86)	-34.67 (134.93)	0.561	0.834
	Success	1.94 (1.18)	1.87 (1.10)	3.66 (83.01)	0.741	
	P_1_	0.560	0.356			
O_^2^_ extraction ratio	Fail	0.30 (0.08)	0.30 (0.09)	1.45 (20.49)	0.895	0.398
	Success	0.29 (0.07)	0.28 (0.07)	-2.65 (21.55)	0.158	
	P_1_	0.589	0.226			
a-vO_2 _difference	Fail	341.64 (85.43)	339.13 (96.45)	1.22 (21.47)	0.868	0.618
	Success	339.51 (94.33)	327.21 (102.02)	-0.89 (24.98)	0.350	
	P_1_	0.925	0.620			
Mod-RER	Fail	1.25 (0.73)	1.82 (1.02)	54.77 (59.57)	<0.001	<0.001
	Success	1.14 (0.58)	1.06 (0.51)	3.32 (63.69)	0.386	
	P_1_	0.503	<0.001			
ΔPaCO_2_	Fail	5.89 (3.35)	5.67 (3.31)	-31.76 (119.4)	0.775	0.855
	Success	6.53 (4.04)	6.12 (3.65)	2.18 (88.63)	0.557	
	P_1_	0.474	0.590			
Δ Mod-RER	Fail	0.16 (0.12)	0.19 (0.15)	5.29 (148.90)	0.194	0.077
	Success	0.16 (0.11)	0.14 (0.09)	-7.24 (95.62)	0.226	
	P_1_	0.933	0.087			

**Figure 2:**
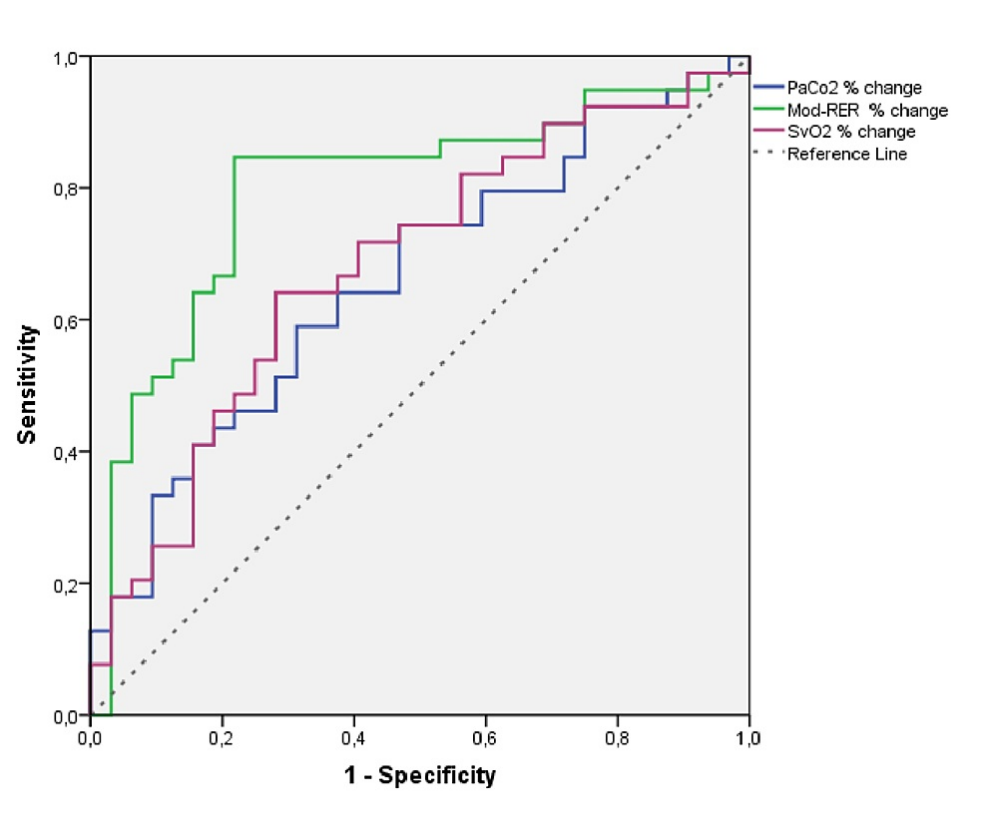
ROC curve for the prediction of outcome from ScvO2%, PaCO2%, and Mod-RER% change ROC: receiver operating characteristic; ScvO2: central venous oxygen saturation; PaCO2: partial pressure of carbon dioxide in arterial blood; Mod-RER: modifying respiratory exchange ratio index

Table 3 presents the results from ROC analysis for prediction of outcome based on ScvO_2_, PaCO_2_, and Mod-RER (Figure 2) changes; AUC was 0.68 (p=0.009) for ScvO_2_ change, 0.66 (p=0.020) for PaCO_2_ change, and 0.80 (p<0.001) for Mod-RER change. The optimal cut-off for ScvO_2_ for change in the outcome prediction was -0.04% (64.1% sensitivity-71.9% specificity). Optimal cut-off for PaCO_2_ change was 6.5% (59% sensitivity- 68.7% specificity). Furthermore, Mod-RER change exhibited the highest sensitivity (84.6%) and specificity (78.1%) with the optimal cut-off of 19.3%. The comparison of AUCs did not reach statistical significance (p=0.106). 

**Table 3:** ROC analyses for the prediction of outcome from ScvO2, PaCO2, and Mod-RER changes AUC: area under curve; PPV: positive predictive value; NPV: negative predictive value; ROC: receiver operating characteristic; ScvO2: central venous oxygen saturation; PaCO2: partial pressure of carbon dioxide in arterial blood; Mod-RER: modifying respiratory exchange ratio index

Change %	AUC	95% CI	P	Optimal cut-off	Sensitivity %	Specificity %	PPV %	NPV %
ScvO_2_	0.68	0.56 - 0.81	0.009	-0.04	64.1	71.9	73.5	62.2
PaCO_2_	0.66	0.53 - 0.79	0.020	6.50	59.0	68.7	69.7	57.9
Mod-RER	0.80	0.69 - 0.91	<0.001	19.30	84.6	78.1	82.5	80.7

When logistic regression analysis in a stepwise method was conducted (Table 4) with dependent variable the weaning trial success, we found that Mod-RER change, ScvO_2_ change, and dementia or anoxic-ischemic encephalopathy were independently associated with the outcome. Specifically, trials in subjects with dementia or anoxic-ischemic encephalopathy had a 95% lower likelihood to succeed, while those with ScvO_2_ change more than -0.04 had a 6.65 times greater likelihood to succeed. Additionally, patients with Mod-RER change<19.3% had 18.33 times greater odds to succeed.

**Table 4:** Results from stepwise logistic regression analysis with dependent variable the outcome Mod-RER: modifying respiratory exchange ratio index; ScvO2: central venous oxygen saturation

	OR (95% CI)	P-value
Mod-RER change (<19.3%)	18.33 (4.43-75.82)	<0.001
ScvO2 change (>-0.04%)	6.65 (1.37-32.21)	0.019
Dementia or anoxic-ischemic encephalopathy		
No (reference)		
Yes	0.05 (0.01 ─ 0.73)	0.029

## Discussion

This study is the first to our knowledge that tests the performance of a modified CPET index as an outcome predictor of extubation in ICU. Our study was conducted on a medical-surgical ICU population with advanced mean patient age (68.4 years) and considerably high Acute Physiology and Chronic Health Evaluation II (APACHE II) score (average 21.2) after a prolonged duration of mechanical ventilation (15 days), with a high incidence of cardiac and respiratory disease. This study showed that a modified CPET index based on RER (Mod-RER) can effectively predict the outcome of a T-piece trial. A significant rise in the values of Mod-RER index, strongly related to anaerobic metabolism, correlated with extubation failure. The proposed index is very easy to measure, doesn’t require any additional equipment, and can be calculated in real time by exploiting easy-to-obtain parameters of blood gas analysis.

Questions remain on why Mod-RER doesn’t perform well in very short T-piece trials (up to 30 minutes) observed in a subgroup analysis based on the duration of the trials. Knowing that venous PO_2_ values (both femoral and mixed venous) fall during exercise [16, 17] while the significant raise of PvCO_2_ is delayed, since it correlates with anaerobic metabolism [9], in short but unsuccessful T-piece trials Mod-RER index probably fail to rise timely. Previous studies reported no difference in the extubation outcome regarding trial duration [24,25], so trials longer than the proposed 30 minutes [1] monitored with Mod-RER could provide us with important outcome information.

Several indices that predict the outcome of extubation have been developed in the past. Marini et al. reported that a maximum inspiratory pressure (Pimax) value of <20-25 cmH_2_O predicts successful weaning [26]. Yang and Tobin prospectively compared P_i_max with the respiratory frequency to tidal volume (f/VT) index in extubation, establishing the “rapid shallow breathing” index as a predictor of weaning failure [27]. Mixed venous oxygen saturation and arterial lactate levels have also been used as weaning indices together with oxygen extraction ratio, oxygen consumption, and delivery [18,19]. Parameters of respiratory mechanics, compliance, and resistance have been tested with significant outcomes [28,29]. In a recent study, Mallat et al. found that changes in ΔPCO_2_ and ScvO2 during spontaneous breathing trials were independently associated with extubation failure and combination analysis of both parameters increased predictability of extubation failure detection [30].

Our study has numerous limitations. First, it lacks well-established theoretical documentation. The modification of a CPET index for ICU patients using partial pressures of gases in arterial and central venous blood seems arbitrary and opposes well-established principles of respiratory and exercise physiology in the name of simplicity. Yet these principles mainly apply to healthy subjects with a status of aerobic metabolism. Our study population was ICU patients with severe co-morbidities, prolonged ICU stays, sometimes with a previous weaning failure, and probably unable to perform even the minuscule work. In such patients, anaerobic metabolism and subsequent lactic acidosis may appear in the very early stages of the trial and affect the accuracy of calculations based on fundamental physiology principles [9]. The second limitation is the extrapolation of data from whole-body exercise studies and application to our population, which underwent a specific form of “exercise” involving only particular muscles (the diaphragm and perhaps thoracic muscles). It was questionable whether fatigue of specific muscles would lead to alterations in pooled blood measurements (i.e. central venous blood). Yet we did observe changes supporting our hypothesis. For example, there were statistically significant lower central venous PO_2_ values at the end of the trial in patients that failed the trial, compared to those that overcame the extubation stress (p=0.03). The third limitation is the small number of T-piece trials studied. The number of extubations was even smaller, yet the information collected from failed T-piece trials was particularly important. A fourth limitation is that Mod-RER index change doesn’t detect the origin of weaning failure, although it certainly indicates reduced cardio-respiratory reserve. Yet, in an era of worldwide increased demand for ICU beds due to the COVID-19 pandemic, we consider such information useful albeit incomplete. Fifth, the inclusion of patients with tracheostomy is a matter of discussion, since we know that the work of breathing is decreased in such patients and this can be a cause of discrepancy in our results. And finally, the fact that we only included T-piece spontaneous breathing weaning trials and no other modes with promising results (such as pressure support spontaneous breathing trials). As we have previously mentioned, we monitored the weaning trials in our ICU, and at the time of the study, this was the only mode of weaning trial in our department.

These are preliminary data on the implementation of a novel prediction tool for extubation outcomes in ICU patients. Further investigation is needed on the effectiveness of the Mod-RER index in specific populations, perhaps patients that fulfill “difficult-to-wean" criteria or after a documented failed weaning trial. Finally, a future trial with a larger sample size, designed on an index-driven decision to extubate the patient or not would be necessary so as to prove its efficacy in safe extubation in difficult-to-wean ICU patients. 

## Conclusions

The weaning procedure in ICU patients is of major importance and critical for the final outcome. Prediction of successful extubation by implementing accurate and easy-to-obtain bedside measurements is tempting and perilous at the same time. In this prospective study, we tested a novel index derived from measurements of partial pressure of oxygen and carbon dioxide in arterial and venous blood samples with quite promising results. Further investigations will determine whether this index could aid a robust prediction of the extubation outcome of ICU patients.
